# Association of Maternal-Clinician Ethnic Concordance With Latinx Youth Receipt of Family-Centered Care

**DOI:** 10.1001/jamanetworkopen.2021.33857

**Published:** 2021-11-10

**Authors:** Cinthya K. Alberto, Jessie Kemmick Pintor, Ana Martínez-Donate, Loni Philip Tabb, Brent Langellier, Jim P. Stimpson

**Affiliations:** 1 Dornsife School of Public Health, Drexel University, Philadelphia, Pennsylvania

## Abstract

**Importance:**

Disparities in medical home provisions, including receipt of family-centered care (FCC), have persisted for Latinx youths in the US.

**Objective:**

To examine the association between maternal-clinician ethnic concordance and receipt of FCC among US-born Latinx youths.

**Design, Setting, and Participants:**

A cross-sectional secondary analysis of data from the Medical Expenditure Panel Survey from January 1, 2010, to December 31, 2017, was conducted. Data analysis was performed from January 6 to February 3, 2020. Latinx youths (age, ≤17 years) born in the US who had a usual source of care and used care in the past year, their Latina mothers (age, 18-64 years), and youths’ health care clinician characteristics (eg, race, ethnicity, and sex) were evaluated using χ^2^ tests and propensity-score matching methods.

**Main Outcomes and Measures:**

Maternal reports on whether their youths’ clinician listened carefully to the parent, explained things in a way the parent could understand, showed respect, and spent enough time with the patient.

**Results:**

There were 2515 US-born Latinx youths with linked maternal characteristics during the study period; 51.67% (95% CI, 48.87%-54.45%) of the youths were male, mean (SD) age was 8.48 (0.17) years (30.86% [95% CI, 28.39%-33.44%] were between ages 5 and 9 years), 61.53% (95% CI, 57.15%-65.74%) had public insurance coverage, and 39.89% (95% CI, 32.33%-47.89%) had mothers who were ethnically concordant with the youths’ medical care clinician. We found that for youths with maternal-clinician ethnic concordance, the probabilities of reporting FCC were significantly higher than they would have been in the absence of concordance: that the medical care clinician listened carefully to the parent (average treatment effect on the treated [ATET], 5.44%; 95% CI, 2.14%-8.74%), explained things in a way the parent could understand (ATET, 4.82%; 95% CI, 1.60%-8.03%), showed respect for what the parent had to say (ATET, 5.51%; 95% CI, 2.58%-8.45%), and spent enough time with the patient (ATET, 5.28%; 95% CI, 1.68%-8.88%).

**Conclusions and Relevance:**

Given the increase of Latinx populations and the simultaneous shortage of underrepresented minority health care clinicians, the findings of this study suggest that increasing the number of clinicians from underrepresented minority backgrounds and ethnic-concordant parental-clinician relationships may help reduce disparities in receipt of medical home provision among US-born Latinx youths.

## Key Points

**Question:**

Is maternal-clinician ethnic concordance associated with youth receipt of family-centered care?

**Findings:**

In this cross-sectional study including 2515 Latinx youths, maternal-clinician Latinx ethnic concordance was found to have had a positive average association with maternal reports of their youths receiving family-centered care, after adjusting for maternal characteristics.

**Meaning:**

The findings of this study suggest that clinicians from underrepresented minority backgrounds contribute to the attenuation of medical home provision disparities among Latinx youths in the US.

## Teaser

This cross-sectional study examines the association between quality of care for Latinx youths and the ethnic concordance of the mothers and clinicians.

## Introduction

In recent decades, there has been an influx of immigrants, asylum seekers, and refugees from Latin America to the US.^1^ Latinx individuals make up the largest ethnic group of youths in the US, and it is projected that by 2050 almost 30% of all youths in the US will be Latinx.^2^ Nearly all (94%) of young Latinx individuals are US born.^3^ At least half of all US-born Latinx youths have at least 1 immigrant parent,^4^ which is associated with social disadvantages compared with US-born Latinx youths with US-born parents, such as a greater likelihood of being uninsured and lacking a usual source of care (eg, health care professional or site).^5,6^

Medical homes are a model of primary care that is patient centered, is comprehensive, recognizes the family as a constant in a child's life, and emphasizes the partnership between health care professionals and families to improve health outcomes.^7^ In addition to access-to-care disparities, US-born Latinx youths face disparities in accessing a medical home. For instance, a study using national data found that only a quarter of Latinx children had a medical home compared with two-thirds of non-Latinx White children.^8^ A family’s experience during a health care visit contributes to and is an important aspect of health care quality and health outcomes.^9^ Third-generation (ie, US-born youths with US-born parents) Latinx youths have yet to reach parity with non-Latinx White youths in reports of the amount of time medical clinicians devote to their care and on clinicians’ sensitivities to their families’ values and customs.^10^ Latinx families headed by immigrant parents also reported receiving less-specific health-related information than families of non-Latinx White youths.^10^ Considering the demographic shifts discussed above, more attention is needed to explore health care experience disparities among US-born Latinx youths based on maternal and clinician characteristics.

Much of the parent-clinician concordance literature among Latinx youths has focused on language.^11,12,13^ Parent-clinician language concordance may be beneficial in communication, but language concordance alone may not equate to reports of higher-quality well-child care, whereas cultural competency has been associated with family-centered care (FCC).^11^ For instance, a clinician speaking Spanish alone may not be associated with the clinician spending more time with the patient, listening more carefully, or respecting familial values and customs—all constructs that are of importance for families. Two cross-sectional studies using regional data have examined the association between racial and ethnic parent-clinician concordance and FCC receipt and did not find that ethnic concordance contributed to ethnic differences in parent reports of youths receiving FCC.^12,13^ To our knowledge, maternal-clinician ethnic concordance among Latinx youths has not been examined since then nor has it been examined using a nationally representative database.

There have been considerable publications on the association of patient-physician racial and ethnic concordance with satisfaction among adults with mixed findings.^14,15,16^ Most recently, a randomized clinical trial observed that clinicians who were Black improved preventive health behaviors among Black patients and reduced the Black-White gap in cardiovascular mortality by 19%.^17^ As the US becomes more racially and ethnically diverse, it faces a shortage of clinicians who identify as racial or ethnic minorities.^18,19^ For instance, among active physicians in the US, only 5% are Black and 5.8% are Hispanic, which does not reflect the proportion of Black (13.4%) and Hispanic (18.5%) individuals in the US population.^20,21^ This shortage has broader implications for the practice of medicine because clinicians from racial and ethnic minority groups are more likely to provide culturally competent care; provide care for underserved, underrepresented minority communities; and work in primary care settings.^21,22,23,24,25^

To our knowledge, the association between maternal-clinician ethnic concordance and youth receipt of FCC among Latinx youths has not been recently examined. We examined the association between maternal-clinician ethnic concordance and receipt of FCC owing to the current clinician shortage in the US and because mothers are more likely than fathers to attend office visits^26^ and respond to surveys on youth health care services.^27^ We used the Andersen Model of Health Services^28^ as a framework and hypothesized that maternal-clinician ethnic concordance would be associated with youth receipt of FCC, which has the potential to reduce disparities in the provision of medical homes for US-born Latinx youths.

## Methods

### Data Source

We obtained data on US-born Latinx youths, their mothers, and the youths’ medical care clinicians from January 1, 2010, to December 31, 2017, from the Medical Expenditure Panel Survey (MEPS) household data. The health insurance eligibility unit identifier in MEPS was used to link maternal characteristics to youth observations, resulting in a sample of 2515 US-born Latinx youths younger than 18 years whose mothers identified as Latina. The Agency for Healthcare Research and Quality administers MEPS, which is drawn from a nationally representative subsample of households that participated in the prior year’s National Health Interview Survey. MEPS is a panel survey that allows for the understanding of how changes in a respondent’s health status, income, and use of services are related and information on a person’s demographic characteristics, access to care, satisfaction with care, and insurance coverage are collected.^29^ The data for this study are publicly available and this study did not involve human participation; thus, the Dornsife School of Public Health, Drexel University, indicated that the investigation did not require institutional review board approval and was exempt from the need for informed consent. This cross-sectional study followed the Strengthening the Reporting of Observational Studies in Epidemiology (STROBE) reporting guideline.^30^

### Measures

Our outcome measure of interest was FCC defined by 4 factors: (1) how often a clinician listened carefully to the parent, (2) how often a clinician explained things in a way the parent could understand, (3) how often a clinician showed respect for what the parent had to say, and (4) how often a clinician spent enough time with a patient. Responses for each of the variables were categorized on an ordinal scale of never, sometimes, usually, or always. Consistent with previous studies,^31,32^ we dichotomized always responses to always, and never, sometimes, and usually responses to not always. These variables were derived from the Consumer Assessment of Healthcare Providers and System survey instrument in MEPS, which is designed to measure the quality of care from a consumer’s perspective.^29^ Parents of youths who have a usual source of care and have received care within the past 12 months are asked to complete the Consumer Assessment of Healthcare Providers and System survey.

The main independent variable was maternal-clinician ethnic concordance, which was defined by the youth’s medical care clinician characteristic variables, such as race and ethnicity, reported by their mothers. Mothers self-reported their own racial and ethnic identity and that of their youths’ clinicians in MEPS.^29^ We considered mothers to be ethnically concordant with the youth’s clinicians if the clinician’s ethnicity was classified as Hispanic in MEPS (ie, concordant). Conversely, we classified youth observations with reports of non-Hispanic clinicians as not ethnically concordant with Latina mothers (ie, nonconcordant).

Predisposing variables we adjusted for included a dichotomized maternal language (English/English, Spanish, or Spanish/other), youth’s clinician’s sex (male or female), maternal marital status (married, divorced/separated, or never married), youth age (0-4, 5-9, 10-13, or 14-17 years), youth sex (male or female), maternal age (18-29, 30-39, 40-49, or 50-64 years), maternal non–US-born status (born in the US: yes or no), and US Census residential region (Northeast, North Central/Midwest, South, or West). Enabling variables included youth insurance coverage status (private, public, or uninsured), maternal insurance coverage status (private, public, or uninsured), and income as a percentage of the federal poverty level (≥400%, 300%-399%, 200%-299%, 100%-199%, or ≤99%). We also included survey year to model time-fixed effects.

### Statistical Analysis

We used Stata, version 15 (StataCorp LLC), for all analyses.^33^ Data analysis was performed from January 6 to February 3, 2020. Descriptive statistics were analyzed using χ^2^ tests to assess differences in predisposing and enabling characteristics by maternal-clinician ethnic concordance. We assessed differences in FCC outcomes by concordance through χ^2^ tests and multivariable logistic regression. Some covariates may be associated with the likelihood of mothers being in concordant relationships; thus, we implemented propensity-score matching in attempts to minimize selection bias into the treatment category (eg, concordance)—a widely used method to estimate treatment outcomes when randomization is not feasible.^34,35^ We used a series of propensity-score matching models to match youths based on the probability of concordance. Specifically, we examined the average treatment effect on the treated (ATET) to estimate the potential association of concordance in youths with mothers who were ethnically concordant with clinicians. The ATET estimates differences in reports of FCC among youths with maternal-clinician concordance and what reports may have been had they not had concordant clinicians. Covariates that were balanced to estimate propensity scores were maternal language (English/English and Spanish or Spanish/other), maternal non–US-born status (born in the US: yes or no), youth insurance coverage status (private, public, or uninsured), and maternal insurance coverage status (private, public, or uninsured). We used 1:1 nearest-neighbor matching, and comparisons, associations, and average effects were considered significant at a 2-sided *P* < .05. All estimates were weighted to reflect the noninstitutionalized population of US-born Latinx youths and adjust for complex survey design.

## Results

In our study sample of US-born Latinx youths, 48.33% (95% CI, 45.55%-51.13%) of the children were girls, 51.67% (95% CI, 48.87%-54.45%) were boys, and the mean (SD) age was 8.48 (0.17) years; 39.86% (95% CI, 32.33%-47.89%) of the sample was ethnically concordant between mothers’ and youths’ medical care clinicians, and 85.91% (95% CI, 79.75%-90.42%) of mothers who were ethnically concordant with their youth’s clinicians spoke Spanish or another language, compared with 69.16% (95% CI, 62.73%-74.92%) of mothers who were not ethnically concordant with their youth’s clinicians (Table 1). Slightly more than a third (33.63%; 95% CI, 28.53%-39.15%) of youths with concordant mothers had a female clinician compared with 45.61% (95% CI, 40.80%-50.49%) of youths with nonconcordant mothers. Most concordant mothers were not born in the US (73.60%; 95% CI, 64.10%-81.31%). Concordant mothers were more likely to report youth uninsurance (3.85%; 95% CI, 2.38%-6.18%) and public insurance coverage (70.94%; 95% CI, 64.64%-76.53%) compared with only 25.21% (95% CI, 19.91%-31.37%) reporting youths having private insurance coverage; noncorcordant mothers were more likely to report private insurance coverage (42.21%; 95% CI, 36.37%-48.27%) and were less likely to report public coverage (55.30%; 95% CI, 49.30%-61.15%) and uninsurance (2.5%; 95% CI, 1.61%-3.84%) for their youths compared with concordant mothers.

**Table 1.** Sample Characteristics^a^

Characteristic	Participants, weighted column % (95% CI)			*P* value^b^
	Total	Maternal-clinician ethnic concordance		
		Not concordant	Concordant	
No.	2515	1512	1003	NA
Weighted %	100	60.14 (52.11-67.67)	39.86 (32.33-47.89)	NA
Predisposing factors				
Maternal language				
English/English and Spanish	24.16 (20.06-28.80)	30.84 (25.08-37.27)	14.09 (9.58-20.25)	<.001
Spanish/other	75.84 (71.20-79.94)	69.16 (62.73-74.92)	85.91 (79.75-90.42)	
Clinician sex				
Female	40.82 (37.13-44.62)	45.61 (40.80-50.49)	33.63 (28.53-39.15)	.002
Male	59.18 (55.38-62.87)	54.39 (49.51-59.20)	66.37 (60.85-71.47)	
Maternal marital status				
Married	65.33 (60.69-69.69)	66.38 (60.90-71.45)	63.77 (56.40-70.54)	.16
Divorced/separated	13.33 (11.19-15.81)	11.08 (8.49-14.35)	16.67 (13.17-20.89)	
Never married	21.34 (17.59-25.64)	22.54 (18.29-27.45)	19.56 (13.67-27.18)	
Youth age, y				
0-4	26.54 (23.59-29.71)	27.59 (23.56-32.01)	24.96 (21.66-28.57)	.12
5-9	30.86 (28.39-33.44)	28.47 (25.41-31.76)	34.46 (31.13-37.95)	
10-13	22.48 (20.43-24.67)	23.50 (20.71-26.54)	20.94 (18.27-23.87)	
14-17	20.12 (17.81-22.65)	20.44 (17.18-24.13)	19.65 (16.69-22.99)	
Youth sex				
Female	48.33 (45.55-51.13)	48.86 (44.89-52.85)	47.53 (42.08-53.05)	.73
Male	51.67 (48.87-54.45)	51.14 (47.15-55.11)	52.47 (46.95-57.95)	
Maternal age, y				
18-29	16.90 (14.56-19.53)	17.78 (14.48-21.64)	15.57 (12.21-19.64)	.25
30-39	48.67 (44.54-52.82)	49.17 (44.02-54.35)	47.92 (41.76-54.15)	
40-49	30.30 (26.70-34.16)	28.20 (23.66-33.22)	33.47 (27.90-39.56)	
50-64	4.13 (3.00-5.65)	4.85 (3.15-7.38)	3.04 (2.10-4.37)	
Mother not born in the US	63.90 (59.39-68.18)	57.48 (51.79-62.97)	73.60 (64.10-81.31)	.006
US Census region				
Northeast	13.12 (9.62-17.64)	15.27 (11.06-20.71)	9.87 (5.83-16.22)	<.001
North Central/Midwest	9.00 (5.94-13.40)	10.27 (6.15-16.65)	7.08 (3.91-12.48)	
South	43.71 (34.60-53.26)	32.74 (26.96-39.10)	60.27 (45.82-73.12)	
West	34.17 (27.04-42.10)	41.72 (34.44-49.38)	22.79 (14.96-33.12)	
Enabling factors				
Youth insurance coverage				
Private	35.43 (31.05-40.07)	42.21 (36.37-48.27)	25.21 (19.91-31.37)	<.001
Public	61.53 (57.15-65.74)	55.30 (49.30-61.15)	70.94 (64.64-76.53)	
Uninsured	3.04 (2.15-4.27)	2.50 (1.61-3.84)	3.85 (2.38-6.18)	
Maternal insurance coverage				
Private	43.91 (39.39-48.54)	49.21 (43.05-55.40)	35.91 (30.75-41.43)	.009
Public	24.45 (20.62-28.72)	22.96 (18.10-28.68)	26.68 (20.60-33.80)	
Uninsured	31.64 (26.30-37.51)	27.82 (22.83-33.44)	37.41 (30.60-44.74)	
Income (% FPL)				
≥400%	9.77 (7.18-13.17)	11.28 (7.65-16.33)	7.50 (4.49-12.29)	.02
300%-399%	7.76 (5.70-10.49)	9.44 (6.50-13.51)	5.22 (2.65-10.03)	
200%-299%	14.09 (11.44-17.25)	15.96 (12.24-20.55)	11.28 (8.02-15.64)	
100%-199%	25.51 (22.09-29.27)	26.65 (22.62-31.10)	23.80 (18.42-30.17)	
≤99%	42.86 (38.59-47.24)	36.67 (32.02-41.59)	52.19 (46.60-57.73)	
Contextual characteristic				
Survey year				
2010-2012	28.97 (25.63-32.56)	28.57 (24.34-33.21)	29.58 (24.93-34.70)	.94
2013-2015	45.12 (41.47-49.13)	45.60 (40.34-50.95)	44.41 (39.41-49.52)	
2016-2017	25.91 (22.32-29.85)	25.83 (20.86-31.52)	26.01 (22.06-30.41)	

Abbreviations: FPL, federal poverty level; NA, not applicable.

^a^ Source: Agency for Healthcare Research and Quality.^29^

^b^ Determined using χ^2^ test.

There were no statistically significant differences in the bivariate association between FCC outcomes and maternal-clinician ethnic concordance (Table 2). Of nonconcordant mothers, 16.20% (95% CI, 12.75%-20.36%) reported that their youth’s clinician did not listen carefully to the parent and 18.32% (95% CI, 16.01%-20.74%) reported that their youth’s clinician did not explain things in a way the parent could understand. In addition, 23.41% (95% CI, 21.04%-25.12%) of nonconcordant vs 20.37% (95% CI, 15.43%-22.97%) of concordant mothers reported that their youth’s clinician did not show respect for what the parent had to say, and 17.81% (95% CI, 15.36%-20.01%) of nonconcordant mothers reported that the clinician did not spend enough time with them vs 14.63% (95% CI, 11.04%-17.11%) who were concordant.

**Table 2.** Descriptive Statistics of Family-Centered Care^a^

Characteristic	Participants, weighted column % (95% CI)			*P* value^b^
	Total	Maternal-clinician ethnic concordance		
		Not concordant	Concordant	
No.	2515	1512	1003	
Weighted %	100	60.14 (52.11-67.67)	39.86 (32.33-47.89)	
Family-centered care				
Clinician listened carefully to parent				
No	14.36 (11.51-17.78)	16.20 (12.75-20.36)	11.60 (8.00-16.51)	.06
Yes	85.64 (82.22-88.49)	83.80 (79.64-87.25)	88.40 (83.49-92.00)	
Clinician explained things in a way the parent could understand				
No	17.08 (14.89-20.28)	18.32 (16.01-20.74)	16.83 (13.44-18.04)	.07
Yes	82.92 (79.34-85.00)	81.68 (76.54-89.77)	83.17 (78.54-86.69)	
Clinician showed respect for what the parent had to say				
No	22.29 (19.84-25.36)	23.41 (21.04-25.12)	20.37 (15.43-22.97)	.06
Yes	77.71 (73.42-80.06)	76.59 (74.05-81.62)	79.63 (76.88-82.69)	
Clinician spent enough time with a person				
No	15.92 (13.44-17.09)	17.81 (15.36-20.01)	14.63 (11.04-17.11)	.07
Yes	84.08 (81.74-86.93)	82.19 (79.71-85.04)	85.37 (76.68-88.16)	

^a^ Source: Agency for Healthcare Research and Quality.^29^

^b^ Determined using χ^2^ test.

Maternal-clinician ethnic concordance was associated with all 4 FCC outcomes among US-born Latinx youths in multivariable models (Table 3). Concordance vs nonconcordance was associated with higher odds of reporting that the clinician listened carefully to the parent (adjusted odds ratio [aOR], 1.71; 95% CI, 1.08-2.69), explained things in a way the parent could understand (aOR, 1.75; 95% CI, 1.07-2.10), showed respect for what the parent had to say (aOR, 1.98; 95% CI, 1.20-2.56), and spent enough time with the patient (aOR, 1.45; 95% CI, 1.12-1.88).

**Table 3.** Logistic Regression Estimation of Maternal-Clinician Ethnic Concordance on Family-Centered Care Components^a^

Characteristic	Family-centered care, aOR (95% CI)			
	Clinician listened carefully to parent	Clinician explained things in a way the parent could understand	Clinician showed respect for what the parent had to say	Clinician spent enough time with patient
**Predisposing factors**				
Maternal-clinician ethnic concordance				
No	1 [Reference]	1 [Reference]	1 [Reference]	1 [Reference]
Yes	1.71 (1.08-2.69)^b^	1.75 (1.07-2.10)^b^	1.98 (1.20-2.56)^b^	1.45 (1.12-1.88)^b^
Maternal language				
English/English and Spanish	1 [Reference]	1 [Reference]	1 [Reference]	1 [Reference]
Spanish/other	0.83 (0.45-1.52)	0.80 (0.47-1.36)	0.66 (0.40-1.09)	0.73 (0.43-1.25)
Clinician sex				
Male	1 [Reference]	1 [Reference]	1 [Reference]	1 [Reference]
Female	1.36 (0.98-2.09)	1.21 (0.88-1.65)	1.19 (0.86-1.66)	1.07 (0.83-1.39)
Maternal marital status				
Married	1 [Reference]	1 [Reference]	1 [Reference]	1 [Reference]
Divorced/separated	1.07 (0.60-1.89)	1.00 (0.67-1.50)	0.73 (0.46-1.17)	1.02 (0.72-1.45)
Never married	1.31 (0.82-2.09)	1.16 (0.80-1.68)	1.03 (0.71-1.47)	1.12 (0.80-1.56)
Youth age, y				
0-4	1 [Reference]	1 [Reference]	1 [Reference]	1 [Reference]
5-9	0.93 (0.59-1.47)	0.84 (0.64-1.10)	0.95 (0.66-1.38)	0.90 (0.67-1.20)
10-13	0.96 (0.61-1.54)	0.88 (0.64-1.22)	0.98 (0.64-1.49)	0.86 (0.62-1.80)
14-17	0.74 (0.42-1.29)	0.61 (0.40-0.91)^c^	0.76 (0.47-1.23)	0.85 (0.57-1.25)
Youth sex				
Male	1 [Reference]	1 [Reference]	1 [Reference]	1 [Reference]
Female	1.09 (0.76-1.57)	1.19 (0.92-1.52)	1.19 (0.88-1.62)	0.97 (0.75-1.26)
Maternal age, y				
18-29	1 [Reference]	1 [Reference]	1 [Reference]	1 [Reference]
30-39	1.65 (0.98-2.77)	1.28 (0.84-1.94)	1.11 (0.76-1.62)	1.45 (0.99-2.12)
40-49	1.12 (0.64-1.95)	1.28 (0.80-2.03)	1.02 (0.65-1.59)	1.38 (0.91-2.10)
50-64	2.62 (0.79-4.98)	1.83 (0.76-4.46)	1.71 (0.70-4.22)	1.97 (1.02-3.80)^c^
Mother born in the US				
Yes	1 [Reference]	1 [Reference]	1 [Reference]	1 [Reference]
No	1.56 (0.92-2.65)	1.27 (0.85-1.91)	1.44 (0.94-2.20)	1.04 (0.71-1.52)
US Census region				
Northeast	1 [Reference]	1 [Reference]	1 [Reference]	1 [Reference]
North Central/Midwest	3.14 (1.36-6.27)^b^	2.87 (1.56-5.30)^d^	1.91 (1.04-3.51)^c^	1.78 (1.06-3.03)^c^
South	2.36 (1.08-4.15)^c^	2.11 (1.24-3.60)^b^	1.66 (0.93-2.99)	1.69 (1.09-2.62)^c^
West	1.55 (0.78-3.10)	1.59 (0.99-2.54)	1.30 (0.79-2.14)	1.20 (0.83-1.75)
**Enabling factors**				
Youth insurance coverage				
Private	1 [Reference]	1 [Reference]	1 [Reference]	1 [Reference]
Public	0.54 (0.30-0.97)^c^	0.64 (0.42-0.96)^c^	0.66 (0.39-1.11)	0.90 (0.59-1.40)
Uninsured	0.50 (0.18-1.38)	1.03 (0.48-2.20)	0.67 (0.27-1.65)	0.68 (0.33-1.41)
Maternal insurance coverage				
Private	1 [Reference]	1 [Reference]	1 [Reference]	1 [Reference]
Public	1.57 (0.74-3.33)	1.27 (0.72-2.22)	1.19 (0.68-2.10)	1.55 (0.97-2.48)
Uninsured	1.05 (0.55-2.01)	0.95 (0.61-1.49)	1.08 (0.63-1.83)	1.02 (0.67-1.55)
Income (% FPL)				
≥400%	1 [Reference]	1 [Reference]	1 [Reference]	1 [Reference]
300%-399%	1.33 (0.26-3.72)	1.25 (0.42-3.73)	0.95 (0.26-3.48)	1.51 (0.59-3.85)
200%-299%	0.83 (0.27-2.51)	0.97 (0.43-2.18)	0.83 (0.27-2.49)	0.84 (0.40-1.76)
100%-199%	0.73 (0.25-2.51)	1.08 (0.48-2.43)	0.79 (0.28-2.22)	0.65 (0.31-1.34)
≤99%	0.48 (0.17-1.41)	0.89 (0.40-1.97)	0.60 (0.21-1.73)	0.48 (0.22-1.03)
**Contextual characteristic**				
Year				
2010-2012	1 [Reference]	1 [Reference]	1 [Reference]	1 [Reference]
2013-2015	2.77 (1.70-4.50)^d^	2.32 (0.99-3.12)	1.76 (1.05-2.97)^c^	1.47 (0.88-2.47)
2016-2017	1.71 (0.98-2.98)	1.89 (0.89-2.65)	1.30 (0.68-2.48)	1.52 (0.84-2.79)

Abbreviation: aOR, adjusted odds ratio.

^a^ Source: Agency for Healthcare Research and Quality.^29^

^b^ *P* < .01.

^c^ *P* < .05.

^d^ *P* < .001.

A box plot of propensity scores before and after matching showed a raw or initial propensity toward nonconcordance with balance after matching (Figure). The ATET findings are presented in Table 4. Propensity score–matching models showed findings similar to multivariable logistic regression results. We found that reports of youth receipt of FCC were higher in concordant vs nonconcordant settings: clinician listened carefully to the parent (ATET, 5.44%; 95% CI, 2.14%-8.74%), explained things in a way the parent could understand (ATET, 4.82%; 95% CI, 1.60%-8.03%), showed respect for what the parent had to say (ATET, 5.51%; 95% CI, 2.58%-8.45%), and spent enough time with the patient (ATET, 5.28%; 95% CI, 1.68%-8.88%).

**Figure.**
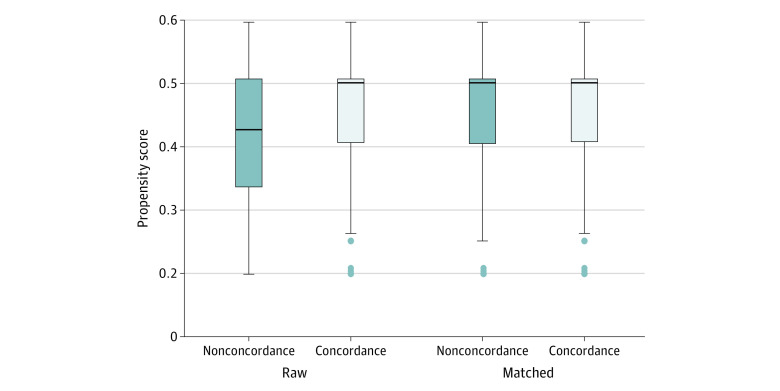
Propensity Scores Toward Concordance vs Nonconcordance Propensity score balance box plot before and after matching. Whiskers represent the 5th and 95th percentiles, boxes represent the 25th and 75th percentiles, dots represent outliers, and horizontal lines inside the boxes are medians.

**Table 4.** ATET of Ethnic Concordance With Maternal-Clinician Ethnic Concordant Relationships^a^

Outcome	ATET, % (95% CI)	*P* value
Clinician listened carefully to parent	5.44 (2.14-8.74)	.001
Clinician explained things in a way the parent could understand	4.82 (1.60-8.03)	.003
Clinician showed respect for what the parent had to say	5.51 (2.58-8.45)	<.001
Clinician spent enough time with a person	5.28 (1.68-8.88)	.004

Abbreviation: ATET, average treatment effect on the treated.

^a^ Source: Agency for Healthcare Research and Quality.^29^

## Discussion

To our knowledge, the association between maternal-clinician ethnic concordance and youth receipt of FCC among US-born Latinx youths has not been examined in nearly 2 decades.^12,13^ We observed an association between maternal-clinician ethnic concordance and reports of youths receiving FCC. To protect against selection bias in concordance, we used propensity score–matching methods. After adjusting for maternal characteristics, we noted that concordance was associated with reports that the youth’s clinician listened carefully to the parent, explained things in a way the parent could understand, showed respect for what the parent had to say, and spent enough time with a patient. Previous research noted that receipt of FCC was associated with health care clinicians eliciting important youth health and developmental information.^36^ For instance, parents of Latinx youths who received FCC had almost twice the odds of clinician elicitation of developmental concerns, which is vital for child development and long-term health outcomes.

Although language use and levels of acculturation among Latinx populations may be important predisposing and enabling factors to examine in health services research, there is a gap in understanding about clinician characteristics in the provision of health care services. Previous literature rooted in frameworks that suggested individuals from minoritized groups have poorer health, health care access, and health services quality because they are not acculturated enough or are limited in their English language abilities contributes to larger population health disparities^37^ and may be perceived to indirectly blame the patient. Furthermore, patient-clinician racial concordance can reduce disparities in infant mortality among Black newborns^38^ by focusing efforts on changing health care system business models among the most marginalized populations,^39^ including Latinx youths. In addition, clinical practices can implement appointment-booking practices to ensure patient-clinician concordance based on race and ethnicity, language, or sex.

Although bivariate analyses of FCC by concordance may appear insignificant, FCC improvements are still meaningful within a population health context. For instance, between 2010 and 2017, the average youth Latinx population in the US was 17 851 822^40^; thus, the change from 1.8% to 5.5% equates to 321 333 to 981 850 US-born Latinx youths receiving FCC. Moreover, the aORs showed an association between concordance and receipt of FCC, and these study findings have health policy and pediatric practice implications. Because Latinx individuals make up the largest racial and ethnic group among all youths in the US,^2^ our study supports the need for more clinicians from underrepresented minority groups. The most recent report from the Association of American Medical Colleges indicates that, among active physicians, only 5.0% are Black and 5.8% are Hispanic.^41^ These proportions of Black and Hispanic clinicians are less than half of the proportion of Black (13.4%) and less than one-third of the proportion of Hispanic (18.5%) individuals in the US population.^20^ Although there was a slight increase in the number of physicians from underrepresented minority groups between 2012 and 2017,^42^ efforts focused on improving these changes have failed to achieve equity in representation. To substantially increase the proportion of clinicians from underrepresented minority backgrounds, we argue that systemic racism must be confronted because it contributes to racial and ethnic educational attainment disparities (eg, equitable public-school funding to achieve high-quality kindergarten through high school education that enables students of racial and ethnic minority groups to competitively apply to health-related higher education and training). It would also be beneficial to increase the 1997 Medicare funding cap for residency training in US medical schools,^43^ reduce university and medical school tuition for underrepresented minorities from lower socioeconomic backgrounds, eliminate the requirement of standardized testing (eg, Medical College Admission Test), and increase antiracist cultural competency training for health care clinicians during medical school and continuing medical education. In addition, federal payers (eg, Medicare, Medicaid/Children's Health Insurance Program) can require clinicians to provide improved care (eg, FCC) for racial and ethnic minority populations.^44^

### Limitations and Strengths

Our study has limitations. First, clinician race and ethnicity were reported by the mothers, which is not optimal and can contribute to information bias, misidentification, and recall bias. For instance, a maternal respondent may have reported their youth’s clinician as being non-Hispanic White, but the clinician may identify themselves as Hispanic. To date, certain sections in MEPS have undergone validity and reliability assessments but have not included the clinician’s race and ethnicity measure,^45,46,47,48^ and work is warranted in assessing this measure further with other data (eg, claims data that include clinician-reported race and ethnicity). Second, we do not have state identifiers and cannot control for state fixed effects that could explain whether there were any improvements in receipt of FCC for youths based on policy changes (eg, Medicaid expansion and increased insurance coverage for youths). Third, although we observed significant findings in maternal-clinician concordance, this study may be limited in power to determine differences in FCC component outcomes by clinician sex, which could also be associated with youth receipt of FCC.^49,50^ Fourth, there may be measurement error in the FCC variables given that mothers may not remember the experience they had when their youths saw a clinician; thus, there may be an underestimation in the number of youths receiving FCC.

Although there are several limitations, the study also has strengths. First, to our knowledge, this was the first study in almost 20 years examining the association of maternal-clinician ethnic concordance and youth receipt of FCC. Second, the bivariate comparisons of FCC by concordance may appear insignificant; however, we believe that although small, these improvements are meaningful within a population health context.

## Conclusions

This cross-sectional study noted an association between maternal-clinician ethnic concordance and receipt of FCC among US-born Latinx youths with Latina mothers. Clinicians from underrepresented racial and ethnic minority backgrounds may contribute to the attenuation of medical home provision disparities among youths in this population of the US. The number of Latinx and Black clinicians has not been commensurate with the population growth of Latinx youths in the US. Increased effort to recruit, train, and hire clinicians from underrepresented minority populations would be beneficial.
